# Test performance and acceptability of self- versus provider-collected swabs for high-risk HPV DNA testing in female-to-male trans masculine patients

**DOI:** 10.1371/journal.pone.0190172

**Published:** 2018-03-14

**Authors:** Sari L. Reisner, Madeline B. Deutsch, Sarah M. Peitzmeier, Jaclyn M. White Hughto, Timothy P. Cavanaugh, Dana J. Pardee, Sarah A. McLean, Lori A. Panther, Marcy Gelman, Matthew J. Mimiaga, Jennifer E. Potter

**Affiliations:** 1 Division of General Pediatrics, Boston Children’s Hospital, Boston, Massachusetts, United States of America; 2 Pediatrics, Harvard Medical School, Boston, Massachusetts, United States of America; 3 Department of Epidemiology, Harvard T.H. Chan School of Public Health, Boston, Massachusetts, United States of America; 4 The Fenway Institute, Fenway Health, Boston, Massachusetts, United States of America; 5 Department of Family & Community Medicine, University of California, San Francisco, San Francisco, California, United States of America; 6 UCSF Center of Excellence for Transgender Health, University of California, San Francisco, San Francisco, California, United States of America; 7 Department of Population, Family, and Reproductive Health, Johns Hopkins School of Public Health, Baltimore, Maryland, United States of America; 8 Chronic Disease Epidemiology, Yale School of Public Health, New Haven, Connecticut, United States of America; 9 Transgender Health Program, Fenway Health, Boston, Massachusetts, United States of America; 10 Beth Israel Deaconess Medical Center, Boston, Massachusetts, United States of America; 11 Department of Behavioral & Social Health Sciences and Epidemiology, Brown University School of Public Health, Providence, Rhode Island, United States of America; 12 Department of Psychiatry & Human Behavior, Alpert Medical School, Brown University, Providence, Rhode Island, United States of America; 13 Center for Health Equity Research (CHER), Brown University, Providence, Rhode Island, United States of America; 14 Department of Medicine, Harvard Medical School, Boston, Massachusetts, United States of America; Bharathidasan University, INDIA

## Abstract

**Background:**

High-risk human papillomavirus (hrHPV) causes virtually all cervical cancers. Trans masculine (TM) people (those assigned female at birth who identify with a gender other than female) have low uptake of conventional cervical cancer screening. Self-collected hrHPV DNA testing has high levels of acceptability among cisgender (non-transgender) females and may support increased cervical cancer screening uptake in TM individuals.

**Objective:**

To assess the test performance and acceptability of self-collected vaginal specimens in comparison to provider-collected cervical swabs for hrHPV DNA detection in TM individuals ages 21–64 years.

**Methods:**

Between March 2015-September 2016, 150 TM participants with a cervix (mean age = 27.5 years; SD = 5.7) completed a one-time study visit comprised of a self-report survey, self-collected vaginal HPV DNA swab, clinician-administered cervical HPV swab, and brief interview on acceptability of clinical procedures. Participants were randomized to complete either self- or provider-collection first to minimize ordering effects. Self- and provider-collected samples were tested for 13 hrHPV DNA types using a DNA Hybridization Assay. The primary outcome variable was the concordance (kappa statistic) and performance (sensitivity, specificity) of self-collected vaginal HPV DNA specimens versus provider-collected cervical HPV swabs as the gold standard.

**Results:**

Of the 131 participants completing both the self- and provider-collected HPV tests, 21 cases of hrHPV were detected by the provider cervical swab (gold standard; 16.0% hrHPV prevalence); 15 of these cases were accurately detected by the self-collected vaginal swab (71.4% concordance) (Kappa = 0.75, 95% Confidence Interval [CI]: 0.59, 0.92; p<0.001). Compared to the provider-collected cervical hrHPV DNA sample (gold standard), the self-collected vaginal hrHPV DNA test demonstrated a sensitivity of 71.4% (95% CI: 0.52, 0.91; p = 0.0495) and specificity of 98.2% (95% CI: 0.96, 1.00; p<0.0001). Over 90% of participants endorsed a preference for the self-collected vaginal swab over provider-collected cervical swab.

**Conclusion:**

Self-collected vaginal swabs are highly acceptable to TM as a means to test for hrHPV DNA. Test performance of this self-collection method for hrHPV detection in TM is consistent with previous studies in cisgender females. Self-collected vaginal swab testing for hrHPV DNA represents a reasonable and patient-centered strategy for primary cervical cancer screening in TM patients unwilling to undergo provider collection of specimens via speculum exam.

## Introduction

Nearly 12,000 cases of cervical cancer are diagnosed each year in the United States (U.S.).[1] Human papillomavirus (HPV) is the most common sexually transmitted infection (STI) in the U.S.,[2] and cervical infection with one of several high-risk strains (hrHPV) is the cause of over 99% of all cervical cancers.[3] Cervical cancer can be prevented with appropriate screening.[4] Current U.S. cervical cancer screening guidelines include cytologic Papanicolaou (Pap) testing every three years for individuals ages 21–65 years who have a cervix regardless of HPV vaccination status, with the recommendation of extending the screening interval to 5 years from ages 30–65 when co-testing for cervical hrHPV infection is performed.[5–8] In 2015, provider-administered cervical hrHPV testing alone, without a Pap test, was proposed as an alternative primary screening methodology,[9] although it has not yet been incorporated into US Preventive Services Task Force (USPSTF) guidelines.[10]

Transgender (trans) people have a gender identity that differs from their sex assigned at birth. Trans masculine (TM) persons, individuals assigned a female sex at birth who identify as a man, male, or another diverse non-binary gender identity on the masculine spectrum, require routine cervical cancer screening as do cisgender (i.e., non-transgender) individuals if a cervix is present. However, neither the 2016 American Congress of Obstetricians and Gynecologists (ACOG) cervical cancer screening recommendations[11] nor the most recent 2012 U.S. Preventive Services Task Force recommendations[5] for cervical cancer screening address screening among TM individuals specifically, although a 2011 ACOG guidance suggests that TM individuals follow the same guidelines.[12] A retrospective chart review study of 350 TM patients with a cervix found that 36% were not current for cervical cancer screening, a significantly higher percentage than cisgender female patients (26%) at the same institution.[13] Disparities are likely greater nationally than among this sample of patients enrolled at a health center specializing in transgender healthcare; it is estimated that overall 25% of transgender individuals avoid accessing preventive healthcare due to discrimination.[14]

A number of factors impact the lower rates of cervical cancer screening among TM. Pelvic exams, including a speculum exam for collecting cervical screening specimens, can cause discomfort and worsen feelings of gender dysphoria among TM.[15] [16] Individual and interpersonal factors including the gendered nature of testing (e.g., “female” waiting rooms, anatomical terminology, and “misgendering” or use of incorrect pronouns), gender dysphoria (distress caused by a nonalignment between gender identity and physical sex characteristics) centered around the genitals, concerns about provider discrimination and insensitivity, or a history of emotional, physical or sexual trauma can contribute to emotional discomfort associated with testing, and pose barriers to screening for TM.[17–23] Long-term testosterone use for masculinization may result in genital atrophy and increased discomfort during the exam.[21, 23] Finally, structural barriers, such as a lack of health insurance or non-coverage of screening due to having a male gender marker on one’s insurance, can prevent access to services.[16]

In past research, TM participants expressed interest in alternative cervical cancer screening methods that do not require a speculum exam.[24, 25] In a survey of 91 transgender men, 50.5% of whom had not had a Pap test within the last 3 years, the majority (57.1%) preferred self-sampling for hrHPV over provider-collected Pap testing, and TM who reported discrimination were more likely to prefer hrHPV self-swabbing.[24] A study of 64 TM completing in-person qualitative interviews and online surveys found that when asked about self-collected swabs for HPV testing as a primary screening method for cervical cancer, the vast majority (94%) were enthusiastic about having self-swabs as a more comfortable and “less invasive” option.[25]

Prior studies examining the use of self-collected vaginal swabs for hrHPV DNA testing among cisgender women have shown high rates of concordance with provider-collected cervical swabs for HPV DNA testing,[26] and have demonstrated sensitivities ranging from 56% to 87% and specificities ranging from 84% to 94% for detection of cervical intraepithelial neoplasia, grade 2/3 using the Dacron swab method.[27–30] A meta-analysis of 6 studies involving self-collection among cisgender female patients found an overall sensitivity of 74% (95% CI: 0.61, 0.84) and specificity of 88% (95% CI: 0.83, 0.92) for detection of HPV infection.[31] Several studies have found primary self-administered hrHPV screening to be highly acceptable among cisgender women and to increase screening rates in underscreened populations.[32–35] Self-collection methods for HPV DNA detection may support increased TM patient engagement in preventive screening and reduce disparities in screening rates, particularly for those who refuse screening using clinician-administered methods.[36]

To our knowledge, there are no published studies of the prevalence of cervical hrHPV infection in TM populations, nor have any investigators examined the relative acceptability of hrHPV self-collection in TM in a study where participants actually received the self-swab and the Pap. As such, we conducted a mixed-methods, biobehavioral study to measure the prevalence of cervical hrHPV infection, and assess the acceptability and clinical performance of self-collected vaginal hrHPV DNA sampling in comparison to provider-collected cervical hrHPV DNA sampling among sexually active TM adults.

## Materials and methods

### Participants and procedures

Between March 2015 and September 2016, 150 TM individuals were enrolled in a study with a single visit.[37] Participants were recruited through a variety of convenience sampling methods including recruitment flyers posted at clinical care sites; facilitated referrals from medical providers and clinical staff; outreach to local organizations and venues frequented by members of the TM community; study information shared through social media, transgender websites and e-mail listserv posts; and via peer-to-peer word of mouth referrals. Interested individuals contacted study staff who administered a brief screening survey in-person or on the phone prior to enrollment. Participants were eligible to participate in the study if they met the following criteria: (1) ages 21 to 64 years; (2) assigned a female sex at birth and now have a masculine spectrum gender identity; (3) have a cervix; (4) have been sexually active within the past 3 years (sexual partner(s) of any gender); (5) able to speak and understand English; (6) willing and able to provide informed consent. Prior HPV vaccination was not grounds for exclusion from the study. Participants were provided with a $100 incentive upon completion of the study activities.

The study was conducted by the Trans Masculine Sexual Health Collaborative at Fenway Health, a federally-qualified community health center that serves the LGBT community in Boston, Massachusetts that is a national leader in transgender public health, clinical care and research.[38] A Community and Provider Task Force comprised of 10 individuals was convened to collaborate with and provide guidance to the investigative team and research staff. The Task Force advised the team on study methods and provided help to ensure that all aspects of the study (e.g., design, instruments, protocols, procedures, recruitment, branding, website, implementation, interpretation and dissemination of findings) were culturally-competent and gender-affirming. All study activities were approved by the Institutional Review Board at Fenway Health.

### Study visit

After consenting to the study, participants first self-administered a survey via electronic tablet on demographics, Pap test utilization history, HPV vaccination history, history of accessing gender affirming medical procedures, healthcare utilization and needs, and sexual behaviors. The survey lasted on average 90 minutes. Following the survey, the clinical portion of the visit was conducted. The order of specimen collection (self- or provider-collected first) was randomized. A randomization table was generated using Statistical Analysis Software version 9.4 (SAS v9.4). Participants were randomized to receive either self- or provider-collection first, and randomization was unblinded. All specimens were collected at the single study visit.

Self-collection of HPV specimens occurred alone in a private exam room or single-stall bathroom, based on participant preference. Trained study staff provided all participants with a written instruction sheet and detailed verbal instructions on self-collection and packaging of specimens. Participants were provided with a hand mirror and latex gloves. Testing swabs and collection tubes were color coded and numbered to prevent confusion. Self-collected vaginal specimens were collected using a sterile polyester-tipped swab Puritan® Medical Products Company LLC, Guilford, ME, USA) inserted approximately two inches into the vaginal canal and rotated in a circular motion for 10–30 seconds. The specimen was then placed in a Cytyc® ThinPrep® solution canister, and tested for 13 high risk HPV types (16, 18, 31, 33, 35, 39, 45, 51, 52, 56, 58, 59, and 68) using a DNA Hybridization Assay via digene Hybrid Capture II® technology (Qiagen Gaithersburg, Inc., Gaithersburg, MD, USA). Testing was conducted by Quest Diagnostics, Marlborough, MA, USA.

Provider-administered sample collection occurred in an exam room by a physician or nurse practitioner. Standardized study practices and fidelity monitoring using audio-recorded visits were implemented to minimize inter-provider variability. Prior to collecting biological specimens, providers conducted a brief pre-exam sexual health history using a standardized script of questions. Cervical specimens were collected using a Medscand® Pap-Perfect® Spatula and Cytobrush Plus (Cooper Surgical, Trumbull, CT, USA) that were deposited into a Cytyc® ThinPrep® solution canister. This sample was tested for (1) abnormal Cytology (classified according to the Bethesda System terminology) by Quest Diagnostics (Marlborough, MA, USA),[39] (2) a DNA Hybridization Assay for 13 high-risk HPV types using digene Hybrid Capture II® technology (Qiagen Gaithersburg, Inc., Gaithersburg, MD, USA), and (3) HPV mRNA E6/E7 using Transcription-Mediated Amplification (TMA) (Aptima®, Hologic, Inc., San Diego, CA, USA). Interim process monitoring raised questions of comparability between provider-collected cervical and self-collected vaginal HPV specimens, given the unknown concordance and performance characteristics of self- and provider-collected vaginal swabs in TM patients. As a result, for the final 53 participants, a clinician-collected vaginal HPV specimen was also collected using a separate sterile polyester-tipped swab (Puritan® Medical Products Company LLC, Guilford, ME, USA) inserted approximately two inches into the vaginal canal and rotated in a circular motion for 10–30 seconds; this specimen was collected with the speculum in place. The specimen was then placed in a Cytyc® ThinPrep® solution canister, and tested for 13 high-risk HPV types (16, 18, 31, 33, 35, 39, 45, 51, 52, 56, 58, 59 and 68) using the DNA hybridization assay via digene Hybrid Capture II® technology (Qiagen Gaithersburg, Inc., Gaithersburg, MD, USA). For participants self-collecting after provider collection, providers removed excess lubricant using an additional cotton swab with ring forceps while withdrawing the speculum.

Other specimens collected included vaginal specimens for trichomonas and bacterial vaginosis, which were performed via the OSOM® Rapid Test (Sekisui Diagnostics LLC, San Diego, CA, USA); vaginal, rectal, and pharyngeal specimens for gonorrhea and chlamydia via the Aptima® Unisex Swab Specimen Collection Kit for Female Endocervical and Male Urethral Swab Specimens (Hologic, Inc., San Francisco, CA, USA) and analysis via APTIMA Combo2® assay (Gen-Probe, Inc., San Diego, CA, USA). Participants were also tested for HIV using the rapid FDA-approved OraQuick® ADVANCE™ HIV-1/2 Antibody Test [sensitivity: 99.6% (98.5–99.9); specificity: 100% (99.7–100)] (OraSure Technologies Inc., Bethlehem, PA, USA) and for syphilis using a Rapid Plasma Reagin (RPR) (Quest Diagnostics, Marlborough, MA, USA) with reflex to titer and confirmatory test.

After completion of specimen collection, a post-interaction questionnaire measuring comfort and satisfaction with the procedure was completed by both providers and participants. Following the collection of biological samples, participants completed a brief qualitative exit interview. Semi-structured interview guides averaged 25 minutes and included questions on experiences with self- and provider-collection, comparison between the two methods, and experiences during the provider encounter. Purposive sampling was utilized to select 50 participants who were offered the option of completing an extended interview for an additional $10. These participants were asked about prior experiences with cervical cancer screening and sexual health screening. Interviews were audio-recorded and transcribed verbatim for analysis.

### Follow-up algorithm for hrHPV+ tests

Participants ages 30–65 testing positive for hrHPV or with abnormal cytology at any age were referred for follow-up according to American Society of Colposcopists and Clinical Pathologist guidelines.[6] As our study sample included individuals ages 21–29 years who would not otherwise be tested for hrHPV, we developed study-specific follow-up recommendations. Individuals 21–24 years testing positive for hrHPV were referred for repeat Pap testing in 3 years if cytology was normal, 1 year if cytology was ASCUS or worse, based on the incidence and natural history of HPV in this age group, and previous research demonstrating that mean time from HPV infection to detection of SIL or CIN was 36 to 43 months.[40] Individuals age 25–29 years testing positive for hrHPV whose Pap cytology was normal were recommended to have repeat Pap testing in 1 year, due to the relatively high incidence of CIN1+ in persons of this age with detectable hrHPV infection and inline with recent 2015 interim clinical guidelines discussing advantages and disadvantages of primary hrHPV testing for cervical cancer screening.[8]

### Statistical analysis

Statistical analyses were conducted in 2017. The primary outcome variables were the concordance and performance of self-collected vaginal HPV DNA specimen versus provider-collected cervical HPV swab as a gold standard. Statistical analysis was conducted using SAS v9.4. A descriptive analysis (frequencies, proportions, means, standard deviations) of demographic and other variables was conducted. Concordance between provider- and self-collection methods was measured by Cohen’s kappa statistic (κ), a measure of the agreement between two methods in excess of that due to chance. The strength of agreement was judged as poor (<0), slight (0 to 0.20), fair (0.21 to 0.40), moderate (0.41 to 0.60), substantial (0.61 to 0.80), and almost perfect (0.81 to 1.00).[41] For test performance, sensitivity and specificity were estimated. Sensitivity was calculated as the number of true positives (self-collected and provider-collected tests both positive for HPV DNA) divided by the number of individuals with HPV (true positives + false negatives). Specificity was calculated as the number of true negatives (self-collected and provider-collected tests negative for HPV DNA) divided by the number of individuals without HPV (true negatives + false positives). Approximate 95% confidence intervals (CIs) were computed for all parameters with an asymptotic variance and critical values from the normal distribution. The hrHPV DNA test conducted on the provider-collected cervical sample was considered the gold standard; all testing results were compared to this method. Given the invasiveness of the exam and the potential for inadequate specimen collection among TM, it was estimated that 10–15% of TM participants would have invalid tests and/or be non-completers of the study protocol. Thus, 150 participants were enrolled to ensure >80% achieved power. As anticipated in sample size calculations, 131 TM contributed data to this analysis (12.7% of the 150 enrolled respondents had invalid tests and/or were non-completers of the full study protocol).

A sensitivity analysis was conducted, based on randomization arm, to assess whether order of testing (provider- or self-collected first) impacted the concordance results. A Breslow-Day test of homogeneity[42] was used to assess the null hypothesis that the odds of having a positive provider cervical hrHPV DNA test result for those with a positive self-swab test result were the same regardless of randomization order. Due to randomization, it was not anticipated that specimen collection order would affect concordance of test results. Based on prior findings suggesting testosterone may affect adequacy of cervical samples,[43] a post-hoc subgroup analysis was conducted to assess whether the effect of any testosterone use as well as duration of use (≥ 5 years vs. < 5 years) impacted concordance results. Exit interviews were audio-recorded and transcribed verbatim. The transcripts were coded and analyzed using techniques from grounded theory.[44] Specifically, transcripts were open-coded by 5 members of the study team for broad analytic themes. The study team worked collaboratively to organize open-coded data into a fixed code structure. This code structure was iteratively refined in a series of team meetings. Once the codebook was finalized, 2 study team members coded all transcripts using Dedoose software.[45] Coded transcripts were compared across coders to ensure consistency of code application and modifications were made to the codebook to improve clarity and reduce redundancies. The authors met frequently throughout the coding process to discuss coding questions and ensure consistent application of codes.

### Data analytic sample

Complete testing data were available for 131 participants. Data from 10 participants who did not complete both self- and provider-collected specimens were excluded (7 participants completed self-collection only, 1 participant completed provider-collection only, 2 participants did not complete either the self- or provider-swab test). Among the remaining 140 participants who received both tests, 9 of the provider samples could not be assayed due to low cellular content. This analysis was therefore restricted to the 131 participants who had available specimens for self-collected vaginal versus provider-collected high-risk HPV cervical DNA.

## Results

### Self-report (Table 1)

**Table 1:** Descriptive characteristics of trans masculine sample (N = 150).

**SOCIO-DEMOGRAPHICS**	**Mean**	**SD**
**Age, continuous**		
Range: 21–50 Years	27.49	5.74
	**N**	**%**
**Race/Ethnicity**		
American Indian or Alaska Native	0	0.0
Asian	9	6.0
Native Hawaiian or Pacific Islander	1	0.7
Black or African American	4	2.7
White	112	74.7
More than one race	23	15.3
Unknown or not reported	1	0.7
**Hispanic/Latino**		
Hispanic or Latino	14	9.3
Not Hispanic or Latino	133	88.7
Unknown or not reported	3	2.0
**Gender Identity**		
Man/Male	43	28.7
Transgender man/FtM	72	48.0
Genderqueer/non-binary	30	20.0
Another Gender Identity	5	3.3
**Education—Highest Level**		
High School or equivalent	14	9.3
Some college (1–3 years)	44	29.3
College graduate (4 year college degree)	46	30.7
Graduate school	46	30.7
**Employment—Current**		
Employed full time	44	29.3
Employed part time	68	45.3
Unemployed	34	22.7
Prefer not to answer	4	2.7
**Student—Current**		
Yes	52	34.7
No	97	64.7
Prefer not to answer	1	0.7
**Income**		
$19,999 or less	45	30.0
$20,000–$39,999	32	21.3
$40,000–$59,999	15	10.0
$60,000–$79,999	16	10.7
$80,000 or more	26	17.3
Don't know	13	8.7
Prefer not to answer	3	2.0
**Insurance**		
No health insurance	4	2.7
Public insurance (Mass Health, Medicaid, Medicare)	45	30.0
Private, school or work insurance	68	45.3
Parent's insurance	31	20.7
Prefer not to answer	2	1.3
**SEXUAL ORIENTATION & SEXUAL PARTNERING**		
**Sexual Orientation**	**N**	**%**
Gay/homosexual/same-gender attracted	13	8.7
Straight/heterosexual	18	12.0
Bisexual	17	11.3
Queer	67	44.7
Pansexual	17	11.3
Questioning/unsure	4	2.7
Asexual	2	1.3
I do not label my sexual orientation	7	4.7
Other	5	3.3
**Number of Partners—Past 36 Months**	**Mean**	**SD**
Range (0 to 50)	6.17	7.5
**Gender of Sexual Partners—Past 36 Months**	**N**	**%**
Cisgender man	84	56.0
Cisgender woman	119	79.3
Transgender man	34	22.7
Transgender woman	29	19.3
Male assigned sex at Birth—Gender non-conforming	14	9.3
Female assigned sex at Birth—Gender non-conforming	42	28.0
**Number of Partners Past 12 Months**	**Mean**	**SD**
Range (0 to 40)	3.15	4.2
**Gender of Sexual Partners—Past 12 Months**	**N**	**%**
Cisgender man	61	40.7
Cisgender woman	91	60.7
Transgender man	23	15.3
Transgender woman	18	12.0
Male assigned sex at birth—Gender non-conforming	8	5.3
Female assigned sex at birth—Gender non-conforming	30	20.0
**MEDICAL GENDER AFFIRMATION**		
**Hormone Use—Lifetime**		
Yes	121	80.7
No	29	19.3
**Time Consistently on Hormones—Lifetime**	**n = 121**	
Less than 6 months	21	17.4
6 months to less than 12 months	17	14.0
12 months to less than 3 years	37	30.6
3 years to less than 5 years	23	19.0
5 years or more	23	19.0
**Hormone Use—Current**		
Yes	113	93.4
No	8	6.6
**Transgender Surgeries**	**n = 150**	
Chest surgery (FTM reconstruction/bilateral mastectomy)	58	38.7
Chest surgery (breast reduction without breast removal)	6	4.0
Facial or neck surgery	8	5.3
Oophorectomy (removal of both ovaries and fallopian tubes)	3	2.0
Partial or Supracervical Hysterectomy (removal of uterus, cervix intact)	2	1.3
Metoidioplasty genital surgery without urethral	1	0.7
**PAP TEST & HPV HISTORY**		
**Pap Test History—Lifetime**	**n = 150**	
Yes	122	81.3
No	27	18.0
Prefer not to answer	1	0.7
**Time Since Last Pap Test**	**n = 122**	
1 year ago or less	45	36.9
More than 1 year ago but not more than 2 years	21	17.2
More than 2 years ago but not more than 3 years	28	23.0
More than 3 years ago but not more than 5 years	17	13.9
More than 5 years ago	11	9.0
**Inadequate Pap—Lifetime**	**n = 150**	
Yes	22	18.0
No	91	74.6
Don't know	9	7.4
**Abnormal Pap—Lifetime**	**n = 122**	
Yes	20	16.4
No	86	70.5
Don't know	15	12.3
Prefer not to answer	1	0.8
**Colposcopy—Lifetime**	**n = 20**	
Yes	9	45.0
No	9	45.0
Don't know	2	10.0
**Biopsy—Lifetime**	**n = 20**	
Yes	3	15.0
No	14	70.0
Don't know	3	15.0
**Heard of HPV**	**n = 150**	
Yes	82	54.7
No	31	20.7
Don't know	37	24.7
**Diagnosed with HPV—Lifetime**	**n = 150**	
Yes	11	7.3
No	135	90.0
Don't know	3	2.0
Prefer not to answer	1	0.7
**Received HPV Vaccine**	**n = 150**	
Yes	84	56.0
No	52	34.7
Don't know	13	8.7
Prefer not to answer	1	0.7
**Number of Doses of HPV Vaccine**	**n = 84**	
1	3	3.6
2	4	4.8
3	72	85.7
Don't know	5	6.0
	**n = 74**	
**Age when First Received HPV Vaccine**	**Mean**	**SD**
Range (age 11 to 34 years)	19.01	4.8

#### Characteristics of the study sample

Participants were mean age 27.4 years (SD 5.8 years, range 21–50); 75.6% identified as white; 14.5% identified as more than one race, 5.3% as Asian, and 3.1% as Black or African American; and 9.9% identified as Hispanic/Latino. Most participants (90.1%) reported completing at least some college or more, and 35.9% reported being currently enrolled as a student. Overall, 75.5% of participants reported some form of employment (30.5% full-time, 45.0% part-time). The most common terms participants used to describe their gender identity were 50.4% Transgender man/FTM, 26.0% Male/Man, and 20.6% Genderqueer/Non-Binary. The majority of the sample (79.4%) reported ever using masculinizing hormones (e.g., testosterone), of which 93.3% (n = 97/104) reported currently taking hormones.

#### Sexual orientation and Gender of Sexual Partner(s)

The most frequently reported sexual orientation was “queer” (42.0%), followed by “straight” (12.2%), “bisexual” (12.2%), “pansexual” (12.2%), and “gay/homosexual/same-gender attraction” (8.4%). Participants reported a mean number of 3.3 sexual partners (standard deviation (SD) 4.4, range 0–40) within the last year. Regarding the gender identity of sex partners within the last year, most (61.1%) had had sex with at least one cisgender woman, followed by 42.7% with a cisgender man, 20.6% with a gender non-conforming/non-binary person assigned a female sex at birth, 16.8% with a transgender man, 13.0% with a transgender woman, and 5.3% with a gender non-conforming/non-binary partner assigned a male sex at birth.

#### Papanicolaou test utilization and HPV vaccination status

The majority (80.2%) reported having received a Pap test at least once in their lifetime. Of those, 15.2% reported having ever received an inadequate result and 15.2% an abnormal Pap result. More than half (55.7%) reported that they had received one or more vaccinations for HPV, while 35.1% reported that they had never been vaccinated, and 8.4% were unsure of their vaccination status. Among the 73 TM who reported one or more vaccinations for HPV, 87.7% completed the vaccination series, 4.1% had two doses, 2.7% one dose, and 5.5% did not know how many doses of vaccine they had received.

### Biological specimens

#### High-risk HPV prevalence (Table 2)

**Table 2:** Prevalence of HPV by diagnostic test (N = 150).

	N = 150	
**Self-Collected Vaginal HPV DNA**	**n**	** %**
Positive	21	14
Negative	126	84
Missing results		
Participant refused test	2	1.3
Other ^a^	1	0.7
**Provider-Collected Cervical HPV DNA (Gold Standard)**		
Positive	21	14
Negative	111	74
Missing results		
Unable to test specimen due to low cellular content	9	6
Participant refused test	2	1.3
Other ^a^	7	4.7
**Provider-Collected Cervical HPV mRNA**		
Positive	19	12.7
Negative	122	81.3
Missing results		
Unable to test specimen due to low cellular content	6	4
Participant refused test	2	1.3
Other ^a^	1	0.7
**Provider-Collected Vaginal HPV DNA**		
Positive	7	4.7
Negative	48	32
Missing results		
Participant refused test	1	0.7
Test not offered ^b^	94	62.7
**Provider-Collected Cervical Cytology**		** **
Normal	111	74
Abnormal		
ASCUS ^b^	5	3.3
LSIL ^c^	4	2.7
Inadequate	27	18
Missing results		
Participant refused test	2	1.3
Other ^a^	1	0.7

Author Notes: Collection of the provider vaginal HPV DNA specimen began with participant 95.

a Other = provider did not collect sample due to low expected cellular content, participant stopped visit due to pain, or lab error.

b ASCUS: Atypical Squamous Cells of Undetermined Significance.

c LSIL: Low-grade Squamous Intraepithelial Lesions.

16.0% of participants (n = 21/131) tested positive for hrHPV types via provider-collected, cervical DNA hybridization assay, which was considered the “gold standard” screening test. A total of 13.0% of participants (n = 17/131) tested positive for hrHPV types via self-collected, vaginal sampled using a DNA hybridization assay. Among the 53 participants who were additionally tested for hrHPV types via provider-collected vaginal specimen, 11.1% (n = 6) tested positive.

#### Cytologic results (Table 2)

Cytologic specimen was adequate for analysis in 105 of 131 (80.2%) participants. Seven participants (6.7% of evaluable samples) had an abnormal result (5 ASCUS, 4 LSIL).

#### Concordance of the self-collected vaginal swab and provider-collected cervical specimen (Table 3)

**Table 3:** Concordance of self-collected vaginal HPV DNA hybridization assay to the provider-collected cervical HPV DNA specimen and to the provider-collected cervical cytology.

** A.**		**Provider-Collected Cervical DNA (n = 131)**			Sensitivity*	Specificity*	PPV*	NPV*	**Kappa**
**Self-Collected** **Vaginal DNA**		Positive	Negative	Total	**95% CI**	**95% CI**	**95% CI**	**95% CI**	**95% CI**
	Positive	15 (11.5%)	2 (1.5%)	17 (13.0%)	71.43%	98.18%	88.24%	94.74%	75.40%
	Negative	6 (4.6%)	108 (82.4%)	114 (87.0%)	0.48, 0.89	0.94, 1.00	0.64, 0.99	0.89, 0.98	0.58, 0.92
	Total	21 (16.0%)	110 (84.0%)	131 (100%)					
** B.**		**Provider-Collected Cervical Cytology (n = 119)**			Sensitivity*	Specificity*	PPV*	NPV*	**Kappa**
**Self-Collected** **Vaginal DNA**		Abnormal ^a^, ^b^	Normal	Total	**95% CI**	**95% CI**	**95% CI**	**95% CI**	**95% CI**
	Positive	6 (5.0%)	15 (12.6%)	21 (17.6%)	66.67%	86.36%	28.57%	96.94%	32.89%
	Negative	3 (2.5%)	95 (79.8%)	98 (82.4%)	0.30, 0.93	0.79, 0.92	0.11, 0.52	0.91, 0.99	0.10, 0.56
	Total	9 (7.6%)	110 (92.4%)	119 (100%)					

* McNemar’s test used due to the dependency of the data. 95% CI based on exact distribution due to small sample size.

^a^ Abnormal = ASCUS or LSIL cervical cytology from provider-administered Pap test.

^b^ Distribution of Abnormal n = 5 ASCUS and n = 4 LSIL.

Author Notes: The primary outcomes findings are based on an analytic sample of 131 participants. Among the 150 enrolled participants, 10 participants did not receive both the vaginal self-swab HPV DNA Hybridization assay and the provider cervical HPV DNA Hybridization assay (gold standard): 7 participants completed the self-swab, but not the provider test; 1 participant completed the provider test, but not the self-swab; and 2 participants did not complete either the provider test or the self-swab test. Of the remaining 140 participants who received both tests, 9 of the provider samples could not be assayed due to low cellular content.

Compared to provider-collected cervical specimens (gold standard), hrHPV DNA hybridization assay via self-collected vaginal specimen had a sensitivity of 71.4% (95% CI: 0.52, 0.91) and a specificity of 98.2% (95% CI: 0.96, 1.00). There was substantial concordance between the sampling methods (κ = 0.75; 95% CI = 0.59, 0.92; p<0.0001).

#### Sensitivity analyses by randomization arm and testosterone (Table 4)

**Table 4:** Sensitivity analyses by randomization arm, current hormone use, and duration of hormone use: Concordance of self-collected vaginal HPV DNA hybridization assay and provider-collected cervical HPV DNA specimen (gold standard).

**A. Randomization Arm:**											
**Provider First**				**Self First**					**Provider First**	**Self First**	**p-value**
	**Provider Cervical HPV DNA**			** **	**Provider Cervical HPV DNA**			**Sensitivity**	77.78%	66.67%	0.66
**Self Vaginal HPV**	**Positive**	**Negative**	**Total**	**Self Vaginal HPV**	**Positive**	**Negative**	**Total**	**Specificity**	96.49%	100.00%	0.50
**Positive**	7	2	9	**Positive**	8	0	8	**PPV**	77.78%	100.00%	0.47
**Negative**	2	55	57	**Negative**	4	53	57	**NPV**	96.49%	92.98%	0.68
Total	9	57	66	Total	12	53	65	**Kappa**	74.30%	76.50%	0.32
**B. Current Hormones:**											
**On Testosterone**				**Not on Testosterone**					**On T**	**Not On T**	**p-value**
** **	**Provider Cervical HPV DNA**				**Provider Cervical HPV DNA**			**Sensitivity**	78.60%	57.10%	0.35
**Self Vaginal HPV**	**Positive**	**Negative**	**Total**	**Self Vaginal HPV**	**Positive**	**Negative**	**Total**	**Specificity**	98.80%	96.30%	0.43
**Positive**	11	1	12	**Positive**	4	1	5	**PPV**	91.70%	80.00%	0.51
**Negative**	3	82	85	**Negative**	3	26	29	**NPV**	96.50%	89.70%	0.17
Total	14	83	97	Total	7	27	34	**Kappa**	82.30%	59.80%	0.20
**C. Duration of Hormone Use:**											
**5+ Years on Testosterone**				**< 5 Years on Testosterone**					**5+ Yrs**	**< 5 Yrs**	**p-value**
** **	**Provider Cervical HPV DNA**				**Provider Cervical HPV DNA**			**Sensitivity**	75.00%	70.60%	1.00
**Self Vaginal HPV**	**Positive**	**Negative**	**Total**	**Self Vaginal HPV**	**Positive**	**Negative**	**Total**	**Specificity**	100.00%	98.00%	1.00
**Positive**	3	0	3	**Positive**	12	2	17	**PPV**	100.00%	85.70%	1.00
**Negative**	1	11	12	**Negative**	5	97	102	**NPV**	91.70%	95.10%	0.50
Total	4	11	15	Total	17	99	119	**Kappa**	81.50%	74.00%	0.60

Author Notes: McNemar’s test used due to the dependency of the data. No statistically significant differences in Sensitivity, Specificity, Positive Predictive Value, Negative Predictive Value by randomization order, current hormone use, longer duration of hormone use. Kappa: The odds of having a Positive provider-collected cervical HPV DNA test result for those with a Positive self-swab HPV DNA test result are the same regardless of randomization order (Breslow-Day test: χ2 = 1.00; df = 1; p = 0.317; aOR = 140.2; CI = 22.8, 1780.0), current hormone use (Breslow-Day test: χ2 = 1.61; df = 1; p = 0.204; aOR = 116.8; CI = 20.8, 1302.0), and being on hormones for 5 years or longer (Breslow-Day test: χ2 = 0.274; df = 1; p = 0.601; aOR = 140.0; CI = 20.3, 1266.0).

Concordance was analyzed by randomization arm (self- versus provider-collection first); the kappa was similar within each strata and overall (77.8% in patients randomized to the provider-collected first arm, 66.7% in self-collected first arm; p = 0.32). There were no statistically significant differences in sensitivity, specificity, PPV, or NPV for provider-collected cervical vs. self-collected vaginal hrHPV DNA specimens by randomization order.

A post-hoc analysis was conducted to assess whether test performance characteristics differed as a function of hormone therapy for medical gender affirmation. No statistically significant differences in sensitivity, specificity, PPV, or NPV were found for provider-collected cervical vs. self-collected vaginal specimens by current testosterone use (p = 0.20) or longer duration of hormone use among those taking hormones (testosterone ≥ 5 years vs. < 5 years; p = 0.60).

#### Concordance of self- and provider-collected vaginal swabs (Table 5)

**Table 5:** Concordance of the provider-collected cervical HPV DNA specimen to the provider-collected vaginal HPV DNA specimen, and of the self-collected vaginal HPV DNA hybridization assay to the provider-collected vaginal HPV DNA specimen.

** A.**		**Provider-Collected Cervical HPV DNA (n = 53)+**			Sensitivity*	Specificity*	PPV*	NPV*	**Kappa**
		Positive	Negative	Total	**95% CI**	**95% CI**	**95% CI**	**95% CI**	**95% CI**
**Provider-Collected Vaginal HPV DNA**									
	Positive	6 (11.3%)	0 (0.0%)	6 (11.3%)					
	Negative	1 (1.9%)	46 (86.8%)	47 (88.7%)	85.71%	100.00%	100.00%	97.87%	91.20%
	Total	7 (13.2%)	46 (86.8%)	53 (100%)	0.42, 1.00	0.92, 1.00	0.54, 1.00	0.89, 1.00	0.74, 1.00
** B.**		**Provider-Collected Vaginal DNA (n = 54)++**			Sensitivity*	Specificity*	PPV*	NPV*	**Kappa**
		Positive	Negative	Total	**95% CI**	**95% CI**	**95% CI**	**95% CI**	**95% CI**
**Self-Collected Vaginal DNA**									
	Positive	6 (11.1%)	1 (1.9%)	7 (13.0%)	85.71%	97.87%	85.71%	97.87%	84.59%
	Negative	1 (1.9%)	46 (85.2%)	47 (87.0%)	0.42, 1.00	0.89, 1.00	0.42, 1.00	0.94, 1.00	0.61, 1.00
	Total	7 (13.0%)	47 (87.0%)	54 (100%)					

Author Notes

+Out of 57 tests, there were 4 missing results in the cross tabs (4 missing cervical DNA results of which 2 were also missing for the provider vaginal).

++Out of 57 tests, there were 3 missing results in the cross tabs (2 provider-collected vaginal tests, 1 self-collected vaginal test).

* McNemar’s test used due to the dependency of the data. 95% CI based on exact distribution due to small sample size.

A total of 53 participants were administered an HPV DNA hybridization assay conducted on a provider-collected vaginal specimen. The DNA test conducted on the provider-collected vaginal specimen, when compared to the same test on the provider-collected cervical specimen (gold standard), had a sensitivity of 85.7% (95% CI: 0.42, 1.00) and specificity of 100.0% (95% CI: 0.92, 1.00). There was 91.2% (95% CI: 0.74, 1.00) concordance between these two tests.

Using the test run on the provider-collected vaginal specimen as the gold standard, the DNA test conducted on the self-collected vaginal specimen had a sensitivity of 85.7% (95% CI: 0.60, 1.00) and a specificity of 97.9% (95% CI: 0.94, 1.00). There was almost perfect concordance between these two tests (kappa = 0.84, 95% CI: 0.61, 1.00; p<0.0001).

### Exit interviews

#### Participant acceptability

In qualitative exit interviews, the self-collected vaginal HPV swab was found to be highly acceptable to TM participants with over 90% endorsing a preference for a self- over provider-collected swab. Positive aspects cited about the experience of self-swabbing included ease, privacy, minimized invasiveness, and a sense of self-empowerment. Several participants noted concerns, such as uncertainty about whether they had performed the procedure correctly, distrust of the accuracy of the test, gender dysphoria triggered by interacting with genitals during self-collection, and difficulty with body positioning or angle of the swab. Participants expressed hope that their comfort with the procedure and their trust in the accuracy of the results it yielded would increase with practice and with clear, comprehensive instructions. Participants recommended developing a video and accompanying written instructions specifically for TM patients to improve self-swab technique among patients. Even when citing concerns, many participants indicated the importance of having alternative cervical cancer screening options for TM individuals. Many respondents indicated that the presence of these alternatives would increase TM healthcare empowerment and uptake of screening, in particular among those who may otherwise never undergo screening.

## Discussion

To our knowledge, this is the first study of the acceptability of self- vs. provider-collected swabs for HPV DNA testing in TM patients wherein patients actually engaged in both tests as part of the study and provided immediate feedback. In this sample of 131 transgender men and other trans masculine individuals undergoing cervical cancer screening by both provider- and self-collected methods, we found a prevalence of high-risk HPV infection by provider cervical sampling of 16%. To our knowledge, these are the first laboratory-confirmed prevalence estimates of high-risk HPV infection in TM individuals; this prevalence is comparable to rates of 10.6% to 23.7% found in cisgender women.[46–49] Our finding of 7% prevalence of abnormal cytology is consistent with previous studies of TM.[43] These findings support the need for cervical cancer screening and HPV vaccination in TM individuals as is recommended for cisgender females.[7, 8, 50, 51]

Self-collected specimens yielded an hrHPV prevalence of 13% and there was substantial concordance (kappa = 75.40, 95% CI: 0.58, 0.92; p<0.001) between the 2 methods. Compared to the hrHPV DNA hybridization assay conducted on a provider-collected cervical specimen, the assay conducted on a self-collected vaginal specimen had a sensitivity of 71% and a specificity of 98%, with an NPV of 95% and a PPV of 88%. This is comparable to study findings of cisgender women which demonstrate a sensitivity of 74% and a specificity of 88% for self-collected vaginal swabs vs. provider-collected specimens.[31] Researchers measuring the performance of self-collected hrHPV testing in cisgender women have consistently supported the use of this screening modality in patients who are otherwise unable or reluctant to undergo Pap testing. Given similar results in this study, we believe that self-collected vaginal hrHPV testing represents an important alternative screening option for TM populations, in particular given that some TM are less likely to be up-to-date on Pap testing relative to cisgender women.[43]

Although cytology alone and hrHPV co-testing remain the screening options recommended in major guidelines in the U.S.,[9, 10] primary high-risk HPV screening is approved by the U.S. Food and Drug Administration as an alternative to current U.S. cytology-based cervical cancer screening methods. Primary hrHPV testing has improved sensitivity compared to cytology; however, false negative hrHPV test results will occur.[9] A primary hrHPV screening strategy shows promise as an alternative approach for those who decline conventional screening, and some data suggests that primary hrHPV screening may be an appropriate first-line cervical cancer screening modality.[8] Several RCTs have found that primary hrHPV screening is at least as effective as cytology when performed at the same screening intervals.[52–54] A large prospective trial of primary hrHPV screening demonstrated improved sensitivity of CIN2 and CIN3 over cytology alone.[52] Research suggests that primary hrHPV testing with a negative result with a 3-year screening interval is at least as effective as a 5-year co-testing strategy. Because the negative predictive value of the test will be even greater in low-prevalence subgroups, this alternative screening strategy may be a particularly good option for low-risk patients; additional research should support the development of a risk prediction tool to aid in clinical decision-making.

Most sexually active individuals will acquire genital hrHPV infection at some point in their lives; however, the majority of HPV infections (90%) will clear on their own without leading to cervical abnormalities, particularly for individuals of younger ages.[2] Over-screening and over-diagnosis are therefore concerns when conducting hrHPV testing as a primary screening approach as compared to Pap testing, as is the potential for excess colposcopy.[55] There is no current consensus for the management of patients undergoing hrHPV–only screening patients who have positive test results, with no clearly validated optimal practice.[8] Research has shown that initial triage of hrHPV positive cisgender female patients with cytology, followed by repeat cytology testing at 12 months, can yield a high negative predictive value and modest colposcopy referral rate, suggesting that this may be the a feasible management strategy.[56] In this study, the triage algorithm for TM individuals testing hrHPV positive was based on age and cytology results (see Methods). Additional research is needed to identify and validate an optimal follow-up algorithm for primary hrHPV screening.

There were no significant differences by testosterone use in clinical performance of the self-collected vaginal hrHPV test as compared to the gold standard. This uniform performance regardless of testosterone use is particularly notable given that TM patients are roughly 10 times more likely to have an inadequate Pap test after 6 months of testosterone therapy.[43] These data suggest that self-swabs have adequate clinical performance to serve as an alternative choice for TM patients who otherwise would not undergo screening by Pap test or who have had issues obtaining an adequate cervical cytology sample.

While clinical performance of the self-collected vaginal swab test was adequate, reduced sensitivity as compared to provider-collected cervical collection is a concern. One potential factor driving reduced sensitivity is inherent to the site of collection (i.e., cervical vs. vaginal). Previous work has shown that oncogenic HPV has particular tropism for the columnar/metaplastic cells of the cervical squamocolumnar junction, rather than the mature squamous epithelium that is present in both ectocervical and vaginal mucosae;[57] as such, vaginal swabs may not reach the optimal site for hrHPV DNA detection. Given that provider-collected vaginal hrHPV DNA swabs also had decreased sensitivity (86%) compared to the provider-collected cervical gold standard, it is likely that a vaginal swab collected by any means is simply not as sensitive as a cervical swab, even when obtained by a technically skilled provider.

A second potential factor, which may be ameliorated, is lack of patient experience in collecting the vaginal sample. Provider-collected vaginal specimens yielded better sensitivity than patient-collected vaginal specimens (86% vs. 71%) when comparing both to the gold standard test, despite the fact that both swabs were collected from the same site. Although this difference did not reach statistical significance, likely to due being underpowered (provider-collected vaginal specimens began with participant 95). This suggests that a technically skilled provider may achieve a more robust sample than a less experienced patient. Sampling accuracy could potentially be increased by refining self-swabbing instructions. Additionally, several self-collection devices and methods have been used and are currently under evaluation, such as cytobrush,[31, 32, 58] tampon,[59, 60] cervical lavage,[61, 62] and dry storage and transport devices;[63, 64] it is possible that they may be less prone to user error, perform better than the self-swab, and warrant future evaluation in TM patients.

Participants experienced a high rate of unsatisfactory cervical Pap results, approximately 20% in our sample. A prior study by our team found that Pap test results were unsatisfactory for evaluation 10.8% of the time among TM patients, compared to only 1.3% of the time for cisgender women.[43] The elevated prevalence of inadequate cytology results observed in this study is likely in part due to the use of carbomer-containing lubricant during the speculum exam, which has been linked previously to a higher rate of inadequate readings.[65] Carbomer-containing lubricant predominately interferes with the processing of liquid cytology samples during the centrifuging process, but does not affect the accuracy of HPV DNA testing among samples that are adequate (communication with Quest Diagnostics on 12/6/2016). Additionally, since swabbing order was randomized, there should not be any differential effect on any one group related to carbomer exposure or lack of exposure.

In considering participant qualitative feedback, it is important to note that findings may be affected by a self-selection bias. TM individuals who volunteered to participate in this study were, by virtue of their decision to volunteer, those who were willing to undergo Pap testing and therefore less averse to receiving healthcare compared to other TM individuals or communities. Individuals with higher levels of Pap testing avoidance, who would benefit most from the option to self-swab, are likely underrepresented in this study. We therefore hypothesize that the benefits of introducing self-collected hrHPV DNA testing as a screening alternative would be even greater at a population level than indicated in this study, unless such individuals are so averse to healthcare or so averse to interacting with their genitals during performance of a self-swab that they are unwilling to engage even in self-collected testing. Additional research is needed to replicate these findings and to reach TM individuals who might not otherwise access cervical cancer screening via provider-patient interactions, yet who may opt to self-swab.

## Conclusions

Given that hrHPV is the primary cause of cervical cancer, screening for hrHPV infection represents an important preventive healthcare strategy. Prevalence of hrHPV in TM individuals in this study is comparable to percentages shown in other populations.[49] TM individuals are indeed at risk for developing cervical cancer and support ACOG recommendations for preventive screening in this population. This study found that self-collected swabs detected fewer cases of hrHPV compared to traditional cervical hrHPV DNA testing conducted by a medical provider, however the negative predictive value approached 95%. The performance characteristics of this self-collection method for hrHPV DNA detection in TM are consistent with previous studies in other natal female populations.[31] Self-swabbing had high acceptability (>90%) in TM individuals as compared to a Pap or provider-collected cervical swab, and therefore has the potential to reduce screening disparities in this population.

For cisgender female patients willing to undergo pelvic examination, current recommended cervical cancer screening modalities include provider-collected cervical cytology alone (ages 21–29), provider-collected cervical cytology/hrHPV DNA co-testing (ages 30–65), and provider-collected cervical hrHPV DNA testing alone (ages 25–65).[5–8] While few consensus groups have made recommendations for TM patients specifically, those that have done so recommend adherence to guidelines for cisgender women.[12] However, data from this study and others[16, 66, 67] show that TM individuals may avoid healthcare for a variety of reasons including fear of discrimination, gender dysphoria, or trauma history. For TM patients who avoid healthcare or who are unwilling or unable to undergo pelvic examination, performance of a vaginal self-swab for hrHPV DNA testing may represent a reasonable, patient-centered, and empowerment-based approach to screening in a comprehensive harm-reduction model of care. Self-collection methods for hrHPV DNA detection may increase TM patient engagement in preventive screening and reduce disparities in cervical cancer screening rates. Additional guidance is needed to establish the safety of this practice given reduced sensitivity of vaginal self-swab relative to traditional Pap tests.
